# Evaluating Microarray-based Classifiers: An Overview

**DOI:** 10.4137/cin.s408

**Published:** 2008-02-29

**Authors:** A.-L. Boulesteix, C. Strobl, T. Augustin, M. Daumer

**Affiliations:** 1 Sylvia Lawry Centre for MS Research (SLC), Hohenlindenerstr. 1, 81677 Munich, Germany; 2 Department of Statistics, University of Munich (LMU), Ludwigstr. 33, 80539 Munich, Germany

**Keywords:** accuracy measures, classification, conditional and unconditional error rate, error rate estimation, validation data, variable selection, gene expression, high-dimensional data

## Abstract

For the last eight years, microarray-based class prediction has been the subject of numerous publications in medicine, bioinformatics and statistics journals. However, in many articles, the assessment of classification accuracy is carried out using suboptimal procedures and is not paid much attention. In this paper, we carefully review various statistical aspects of classifier evaluation and validation from a practical point of view. The main topics addressed are accuracy measures, error rate estimation procedures, variable selection, choice of classifiers and validation strategy.

## 1 Introduction

In the last few years, microarray-based class prediction has become a major topic in many medical fields. Cancer research is one of the most important fields of application of microarray-based prediction, although classifiers have also been proposed for other diseases such as multiple sclerosis (Bomprezzi et al. 2003). Important applications are molecular diagnosis of disease subtype and prediction of future events such as, e.g. response to treatment, relapses (in multiple sclerosis) or cancer recidive. Note that both problems are usually treated identically from a statistical point of view and related from a medical point of view, since patients with different disease subtypes also often have different outcomes.

Let us consider a standard class prediction problem where expression data of *p* transcripts and the class information are available for a group of *n* patients. From a statistical point of view, patients are *observations* and *transcripts* are *variables*. Note that a particular gene might be represented several times. To avoid misunderstandings, we prefer the statistical term “variable” to the ambiguous term “gene”. In microarray studies, the number of variables *p* is huge compared to *n* (typically, 5000 ≤ *p* ≤ 50000 and 20 ≤ *n* ≤300), which makes standard statistical prediction methods inapplicable. This dimensionality problem is also encountered in other fields such as proteomics or chemometrics. Hence, the issues discussed in the present article are not specific to microarray data. The term *response class* refers to the categorical variable that has to be predicted based on gene expression data. It can be, e.g. the presence or absence of disease, a tumor subtype such as ALL/AML (Golub et al. 1999) or the response to a therapy (Ghadimi et al. 2005; Rimkus et al. 2008). The number of classes may be higher than two, though binary class prediction is by far the most frequent case in practice.

Note that gene expression data may also be used to predict survival times, ordinal scores or continuous parameters. However, class prediction is the most relevant prediction problem in practice. The interpretation of results is much more intuitive for class prediction than for other prediction problems. From a medical point of view, it is often sensible to summarize more complex prediction problems such as, e.g. survival prediction or ordinal regression as binary class prediction. Moreover, we think that the model assumptions required by most survival analysis methods and methods for the prediction of continuous outcomes are certainly as questionable as the simplification into a classification problem. However, one has to be aware that transforming a general prediction problem into class prediction may lead to a loss of information, depending on the addressed medical question.

Beside some comparative studies briefly recalled in Section 2, several review articles on particular aspects of classification have been published in the last five years. For example, an extensive review on machine learning in bioinformatics including class prediction can be found in Larranaga et al. (2006), whereas Chen (2007) reviews both class comparison and class prediction with emphasis on univariate test statistics and model choice from the point of view of pharmacogenomics. Asyali et al. (2006) gives a wide overview of class prediction and related problems such as data preparation and clustering. Synthetic guidelines for good practice in microarray data analysis including class prediction can be found in Simon et al. (2003); Dupuy and Simon (2007). The latter also gives a critical overview of cancer research articles published in 2004.

In contrast to all these, the present article focuses specifically on the statistical evaluation of microarray-based prediction methods. After a brief overview of existing classification methods in Section 2, measures of classification accuracy including error rate, sensitivity and specificity as well as ROC-curve analysis are addressed in Section 3. Section 4 reviews different evaluation strategies such as leaving-one-out cross-validation or bootstrap methods from a technical point of view, whereas Section 5 gives guidelines for practical studies. An overview of software for microarray-based class prediction in the R system for statistical computing (R Development Core Team, 2006) is given in the appendix.

## 2 Overview of Existing Classifiers

### Coping with high-dimensional data

There exist a variety of classification methods addressing exactly the same statistical problem. Several classifiers have been invented or adapted to address specifically prediction problems based on high-dimensional microarray data. Class prediction can also be addressed using machine learning approaches. The aim of this section is to provide a concise overview of the most well-known classification approaches rather than an exhaustive enumeration. In contrast to other authors, we organize this overview with respect to the scheme used to handle high-dimensionality and not to the classifier itself. From this perspective, methods for handling high-dimensional data can basically be grouped into three categories: approaches based on (explicit) variable selection, procedures based on dimension reduction and methods performing intrinsic variable selection. It should be noted that the three mentioned types of approaches for handling high dimensional data can also be combined with each other. For instance, variable selection may be performed prior to dimension reduction or before applying a method handling more variables than observations.

#### Variable selection

The most intuitive approach consists of first selecting a small subset of variables and then applying a traditional classification method to the reduced data set. By traditional methods, we mean well-known statistical methods handling a rather limited number of variables, such as discriminant analysis methods reviewed and compared by Dudoit et al. (2002) including linear and quadratic discriminant analysis or Fisher’s linear discriminant analysis, classical logistic regression or *k*-nearest-neighbors. In principle, the latter could be applied to a high number of variables but performs poorly on noisy data.

Many variable selection approaches have been described in the bioinformatics literature. Overviews include the works by Stolovitzky (2003) and Jeffery et al. (2006). The methods applied can be classified as *univariate* and *multivariate* approaches. Univariate approaches consider each variable separately: they are based on the marginal utility of each variable for the classification task. Variables are ranked according to some criterion reflecting their association to the phenotype of interest. After ranking, the first variables of the list are selected for further analysis. Many criteria are conceivable, for instance usual test statistics like Student’s t-statistic or nonparametric statistics such as Wilcoxon’s rank sum statistic. Further non-parametric univariate criteria include more heuristic measures such as the TnoM score by Ben-Dor et al. (2000). Some of the nonparametric univariate approaches are reviewed by Troyanskaya et al. (2002). The t-statistic, the Mann-Whitney statistic and the heuristic signal-to-noise ratio suggested by Golub et al. (1999) are the most widely-used criteria in practice (Dupuy and Simon, 2007).

In the context of differential expression detection, several regularized variants of the standard t-statistic have been proposed in the last few years. They include, e.g. empirical Bayes methods (Smyth, 2004). An overview can be found in Opgen-Rhein and Strimmer (2007). Although empirical Bayes methods are usually considered as univariate approaches, they involve a multivariate component in the sense described below, since the statistic of each variable is derived by borrowing information from other variables.

Univariate methods are fast and conceptually simple. However, they do not take correlations or interactions between variables into account, resulting in a subset of variables that may not be optimal for the considered classification task. This is obvious in the extreme case where, say, the 10 first variables correspond to the same transcript, yielding a strong correlation structure. It is then suboptimal to select these 10 redundant variables instead of variables with a worse univariate criterion value but giving non-redundant information.

Multivariate variable selection approaches for microarray data have been the subject of a few tens of rather theoretical articles. They take the preceding argument seriously that the subset of the variables with best univariate discrimination power is not necessarily the *best subset of variables*, due to interactions and correlations between variables. Therefore, multivariate variable selection methods do not score each variable individually but rather try to determine which combinations of variables yield high prediction accuracy. A multivariate variable selection method is characterized by i) the criterion used to score the considered subsets of variables and ii) the algorithm employed to search the space of the possible subsets, an exhaustive enumeration of the 2*^p−^*^1^ possible subsets being computationally unfeasible. Scoring criteria can be categorized into *wrapper criteria*, i.e. criteria based on the classification accuracy or *filter criteria* that measure the discrimination power of the considered subset of variables without involving the classifier, for instance the Mahalanobis distance well-known from cluster analysis (which can roughly be seen as multivariate t-statistic).

There have also been various proposals regarding the search algorithms. Some methods, which could be denoted as “semi-multivariate” restrict the search to pairs of variables (Bo and Jonassen, 2002) or subsets of low-correlated and thus presumably non-redundant variables derived from the list of univariately best variables (Jäger et al. 2003). In contrast, other authors seek for globally optimal subsets of variables based on sophisticated search algorithms such as molecular algorithms (Goldberg, 1989) applied to microarray data by, e.g. Ooi and Tan (2003).

Note that most multivariate variable selection methods take only correlations between variables but not interactions into account, depending on the considered criterion used to score the variable subsets. The recent method suggested by Diaz-Uriarte and de Andrés (2006) based on random forests (Breiman, 2001) is one of the very few methods taking interactions into account explicitly. Potential pitfalls of multivariate methods are the computational expense, the sensitivity to small changes in the learning data and the tendency to overfitting. This is particularly true for methods looking globally for good performing subsets of variables, which makes semi-multivariate methods preferable in our view. Note that, from a technical point of view, univariate variable selection methods, which select the top variables from a ranked list, may be seen as a special case of multivariate selection, where the candidate subsets are defined as the subsets formed by top variables.

In pattern recognition another interesting framework that has been suggested for variable selection is the rough set approach of Pawlak (1991). In this framework, concepts that are relevant for variable selection issues are the so-called *reducts* and the *core* of a data table, that can be outlined as follows. Subsets of variables that are sufficient to maintain the structure of equivalence classes (i.e. to distinguish groups of observations that share the same values of a set of variables) form the reduct. In a given data set, several reducts may equally well represent the structure of equivalence classes. However, a (potentially empty) subset of variables is included in all reducts. This so-called core contains those variables that cannot be removed from the data set without loosing information. In addition to the reducts and core based on the structure of the variables, the relative reducts and the relative core also incorporate the response class membership by means of rough sets. They are thus interesting concepts for variable selection issues in classification problems. This approach can be considered as a multivariate variable selection method because it reflects correlations within the data matrix and can be extended to cover interaction effects (Li and Zhang, 2006). However, Kohavi and Frasca (1994) show in a study on simulated and real data that the subset of features that is optimally suited for classification is not necessarily a relative reduct (i.e. does not necessarily include the relative core) but note that using the relative core as a starting point for, e.g. stepwise variable selection may be more promising (cf also Swiniarski and Skowron, 2003; Zhang and Yao, 2004, for overviews on rough set approaches for variable selection and recent advancements).

#### Dimension reduction

A major shortcoming of variable selection when applied in combination with classification methods requiring the sample size *n* to be larger than the number *p* of variables is that only a small part of the available information is used. For example, if one applies logistic regression to a data set of size *n* = 50, the model should include at most about 10 variables, which excludes possibly interesting candidates. Moreover, correlations between variables are not taken into account and can even pose a problem in model estimation, the more as gene expression data are known to be highly correlated. An option to circumvent these problems is dimension reduction, which aims at “summarizing” the numerous predictors in form of a small number of new components (often linear combinations of the original predictors). Well-known examples are Principal Component Analysis (PCA), Partial Least Squares (PLS, Nguyen and Rocke, 2002; Boulesteix, 2004; Boulesteix and Strimmer, 2007) and its generalizations (Fort and Lambert-Lacroix, 2005; Ding and Gentleman, 2005), or the Independent Component Analysis (ICA) motivated approach to classificatory decomposition (Smolinski et al. 2006). A concise overview of dimension reduction methods that have been used for classification with microarray data is given in Boulesteix (2006).

After dimension reduction, one can basically apply any classification method to the constructed components, for instance logistic regression or discriminant analysis. However, as opposed to the original genetic or clinical variables, the components constructed with dimension reduction techniques themselves may not be interpretable any more.

#### Methods handling a high number of variables directly

Instead of reducing the data to a small number of (either constructed or selected) predictors, methods handling large numbers of variables may be used. Preliminary variable selection or dimension reduction are then unnecessary in theory, although often useful in practice in the case of huge data sets including several tens of thousands of variables. Methods handling a high number of variables (*p* ≫ *n*) directly can roughly be divided into two categories: statistical methods based on penalization or shrinkage on the one hand, and approaches borrowed from the machine learning community on the other hand. The first category includes, e.g. penalized logistic regression (Zhu, 2004), the Prediction Analysis of Microarrays (PAM) method based on shrunken centroids (Tibshirani et al. 2002), Support Vector Machines (SVM) (Vapnik, 1995) or the more recent regularized linear discriminant analysis (Guo et al. 2007). Such methods usually involve one or several penalty or shrinkage parameter(s) reflecting the amount of regularization.

Ensemble methods based on recursive partitioning belong to the second category. They include for example bagging procedures (Breiman, 1996) applied to microarray data by Dudoit et al. (2002), boosting (Freund and Schapire, 1997) used in combination with decision trees by Dettling and Buhlmann (2003), BagBoosting (Dettling, 2004) or Breiman’s (2001) random forests examined by Diaz-Uriarte and de Andrés (2006) in the context of variable selection for classification. These methods may be easily applied in the *n* < *p* setting. However, most of them become intractable when the number of features reaches a few tens of thousands, as usual in recent data sets. They should then be employed in combination with variable selection or dimension reduction.

Methods handling a high number of variables can be seen as performing intrinsic variable selection. Shrinkage and penalization methods allow to distinguish irrelevant from relevant variables through modifying their coefficients. Tree-based ensemble methods also distinguish between irrelevant and relevant variables intrinsically, through variable selection at each split.

This somewhat artificial splitting into four categories (variable selection, dimension reduction, regularization methods and machine learning approaches) may inspire the choice of candidate classifiers in practical data analysis. Although our aim is definitely not to rate methods, let us sketch a possible approach inspired from the categories outlined above. Since it is in general recommendable to use methods of different nature, a possible combination would be: i) a simple discriminant analysis method such as Linear Discriminant Analysis (LDA) combined with variable selection, ii) a dimension reduction technique, for instance the supervised PLS approach, iii) some regularization techniques, for instance *L*_1_ or *L*_2_ penalized regression or Support Vector Machines and iv) an ensemble method such as random forests.

### Comparison studies

Prediction methods have been compared in a number of articles published in statistics and bioinformatics journals. Some of the comparisons are so-to-say neutral, whereas others aim at demonstrating the superiority of a particular method. Neutral comparison studies include Dudoit et al. (2002); Romualdi et al. (2003); Man et al. (2004); Lee et al. (2005); Statnikov et al. (2005). Comparison of different classification methods can also be found in biological articles with strong methodological background (e.g. Natsoulis et al. 2005). Most of these studies include common “benchmark” data sets such as the well-known leukemia (Golub et al. 1999) and colon (Alon et al. 1999) data sets. Table 2 (Appendix B) summarizes the characteristics and results of six published comparison studies, which we took as neutral, because they satisfy the following criteria:

- The title includes explicitly words such as “comparison” or “evaluation”, but no specific method is mentioned, thus excluding articles whose main aim is to demonstrate the superiority of a particular (new) method.
- The article has a clear methodological orientation. In particular, the methods are described precisely (including, e.g. the chosen variant or the choice of parameters) and adequate statistical references are provided.
- The comparison is based on at least two data sets.
- The comparison is based on at least one of the following evaluation strategies: cross-validation, Monte-Carlo cross-validation, bootstrap methods (see Section 4).

However, even if those criteria are met, optimistically biased results are likely to be obtained with the method(s) from the authors’ expertise area. For example, authors are aware of all available implementations of that method and will quite naturally choose the best one. They may also tend to choose the variable selection method (e.g. *t*-test or Mann-Whitney test) according to their previous experience of classification, which has been mostly gained with this particular method. Similarly, an inexperienced investigator might overestimate the achievable error rate of methods involving many tuning parameters by setting them to values that are known to the experts as suboptimal.

### The connection between classifiers and variable selection

When performed as a preliminary step, e.g. for computational reasons, variable selection should be seen as a part of classifier construction. In particular, when a classifier is built using a *learning* data set and tested subsequently on an independent *test* data set, variable selection must be performed based on the learning set only. Otherwise, one should expect nonnegligible positive bias in the estimation of prediction accuracy. In the context of microarray data this problem was first pointed out by Ambroise and McLachlan (2002). Although it is obvious that test observations should not be used for variable selection, variable selection is often (wrongly) carried out as a “preliminary” step, especially when classification accuracy is measured using leave-one-out cross-validation. Even if performing *t*-tests or Wilcoxon tests *n* × *p* times becomes a daunting task when *p* reaches several tens of thousands, preliminary variable selection using all *n* arrays and leaving no separate test set for validation should definitively be banished. Bad practice related to this aspect has probably contributed to much “noise discovery” (Ioannidis, 2005).

A further important connection between classifiers and variable selection is the use of classifiers to evaluate the influence of single variables on the response class *a posteriori*. Parametric models, such as the logistic regression model, provide parameter estimates for main effects and interactions of predictor variables that can be interpreted directly for this purpose. However, the modern nonparametric approaches from machine learning, e.g. random forests, also provide variable importance measures that can be used not only for the preselection of relevant variables (Diaz-Uriarte and de Andrés, 2006) but are also a means of evaluating the influence of a variable both individually and in interactions on the response. Random forest variable importance measures have thus become a popular and widely used tool in genetics and related fields. However, when the considered predictor variables vary in their scale of measurement or their number of categories, as, e.g. when both genetic and clinical covariates are considered, the computation of the variable importance can be biased and must be performed differently (Strobl et al. 2007).

## 3 Measures of Classification Accuracy

We have seen in the previous section that in large-scale association studies classification can either be conducted with previous variable selection, dimension reduction, or with special classification methods that can deal with small *n* large *p* problems by intrinsically performing variable selection. However, these methods are very diverse, both in their methodological approach and their statistical features. In the following, we review concepts that allow to evaluate and compare all these different strategies and models and are adaptable to special needs of investigators, e.g. if asymmetric mis-classification costs are supposed to be modelled.

### Error rate

We consider the random vector **X** ∈ℝ *^p^* and the random variable *Y* ∈ {0, …, *K –* 1} giving the “class membership”. Let **F** denote the joint distribution function of **X** and *Y*.A *classifier* is a function from ℝ *^p^* to {0, …, *K –* 1} that assigns a predicted class to a vector of gene expressions corresponding to a patient:

(1) $$\begin{array}{c} C:R^{p}\to \{0,\ldots ,K-1\}\\ X\to \hat{Y}, \end{array}$$

where **X** denotes the *p*-vector giving the gene expression levels of the *p* considered variables for one patient and *Ŷ* is his or her predicted class. If the joint distribution **F**(**X**, *Y* ) of the gene expressions **X** and the class membership *Y* were known, one could use it to construct the Bayes classifier

(2) $$C_{Bayes}(X)=\text{arg}\;\underset{k}{\text{max}}\;P(Y=k|X),$$

by deriving the posterior distribution *P*(*Y |* **X**) of the response class given the gene expressions **X**. The Bayes classifier *C_Bayes_* based on the true, but unfortunately unknown, distributions minimizes the theoretical error rate, i.e. the probability of classifying into the wrong class:

(3) $$\begin{array}{l} Err(C)=P_{F}(C(X)\neq Y)\\ =E_{F}\left(I(C(X)\neq Y)\right). \end{array}$$

Note that this and all following definitions of error rates are appropriate in the case of unordered response classes only. For ordinal response classes it may be desirable that misclassification in a more distant class affects the error term more severely than misclassification in a neighboring class, which could be modelled via pseudo-distances serving as weights in the computation of the error rate. For classifiers that return class probabilities instead of predicted class membership, such as Bayesian methods but also some versions of recursive partitioning, the difference between the predicted class probability and the true class membership can be computed, e.g. by the Brier Score (i.e. the quadratic distance, see, e.g. Spiegelhalter, 1986, for an introduction).

Since the theoretical joint distribution **F** is always unknown in real data analysis, the classifier has to be estimated from an available data set. Moreover, once a classifier is constructed, its error rate also has to be estimated from some available data. Hence, the estimation of the error rate of a classification method involves two estimation components. Suppose we have a data set including *n* patients whose class membership has been determined independently of gene expression data, e.g. by clinical examination. The available data set **D** = (**d**_1_, *…,* **d***_n_*) consists of *n* identically distributed independent observations **d***_i_* = (*y_i_*, **x***_i_*), where *y_i_* ∈ {0, *…, K–*1} denotes the class membership and **x***_i_* = (*x_i_*_1_ , *…, x_ip_*)*^T^* the *p*-vector of gene expressions of the *i*-th patient.

The data set used to construct (i.e.“learn”) a classifier is usually denoted as “training” or “learning” data set. In this article, we use the term *learning data*. Let **l** = (*l*_1_, *…, l_L_*) denote the indices of patients included in the learning data set and **Dl** = (**d***l*_1_, *…,* **d***l_L_*) the corresponding data set, where *L* is the number of observations in **l**. In practice, there are several ways to define **l** and **t**, see Section 4. A *classification method* takes the learning data set **Dl** as input and learns a classifier function *C* as defined in Eq. (1). From now on, *C*_**D_1_**_*^M^* denotes the classifier learnt from the data set **Dl** using the classification method *M*. Examples of classification methods are, e.g.“SVM with linear kernel without preliminary variable selection” or “linear discriminant analysis with the 20 best variables according to the t-test”.

In practical studies, investigators are often interested in the true error rate of a classifier built with all the available observations:

(4) $$Err\left(C_{D}^{M}\right),$$

where **D** is considered as fixed, hence the term *conditional error rate*. However, **D** can also be considered as random. The *unconditional* (or *expected*) true *error rate* is defined as

(5) $${ɛ}_{F^{n}}^{M}=E_{F^{n}}\left(Err\left(C_{D}^{M}\right)\right),$$

where **F***^n^* describes the multivariate distribution of **D** based on **F**(**X**, *Y* ). The unconditional error rate ε_**F**^*n*^_*^M^* depends only on the classification method *M*, the size *n* of the used data set and the joint distribution **F** of **X** and *Y* , but not on the specific data set **D**. We use the notation ε instead of *Err* to outline the difference between conditional and unconditional error rate. Few articles distinguish between the two. However, the relative performance of error estimation methods may depend on whether one considers the conditional or unconditional error rate, see Section 4 for some examples.

### Estimating the error rate

Suppose we use a learning set to construct the classifier *C^M^*_**D**_**l**__. The joint distribution function **F** being unknown, the true conditional error rate

(6) $$Err\left(C_{D_{1}}^{M}\right)=E_{F}\left(I\left(Y\neq C_{D_{1}}^{M}(X)|D_{1}\right)\right)$$

of this classifier is also unknown and has to be estimated based on available test data. Similarly to **l** above, collecting the indices corresponding to the learning data set, we consider the *T*-vector **t** = (*t*_1_, *…, t_T_*) giving the indices of test observations and **Dt** = (**d**_*t*_1__, *…,* **d**_*t_T_*_) the corresponding data set. The estimator of the error rate of *C* based on **Dt** is then given as

(7) $$\hat{Err}\left(C_{D_{1}}^{M},{\text{D}}_{t}\right)=\frac{1}{T}\sum_{i=1}^{T}I\left(y_{t_{i}}\neq C_{D_{1}}^{M}\left({\text{x}}_{t_{i}}\right)\right),$$

where **x***_t_i__* = (*x_t_i__*_1_, *…, x_t_i_p_*) is the *p*-vector giving the gene expressions for the *t_i_*-th observation. Note that, in simulations, the learning set **Dl** can be varied and the test data set **Dt** may be virtually as large as computationally feasible, thus providing an accurate estimation of *Err* (*C*_**D**_**1**__*^M^*).

### Sensitivity and specificity

Using the error rate as defined in Eq. (6), one implicitly considers all misclassifications as equally damaging. In practice, the proportion of misclassified observations might not be the most important feature of a classification method. This is particularly true if one wants to predict therapy response. If a non-responder is incorrectly classified as responder, possible inconveniences are the potentially severe side-effects of a useless therapy and—from an economic point of view—the cost of this therapy. On the other hand a responder who is incorrectly classified as nonresponder may be refused an effective therapy, which might lead to impairment or even death.

In the medical literature, these two different aspects are often formulated in terms of sensitivity and specificity. If *Y* = 1 denotes the condition that has to be detected (for instance responder to a therapy), the *sensitivity* of the classifier is the probability *P*(*C*_**D**_**1**__*^M^*(**X**) = 1 | *Y* = 1) of correctly identifying a responder. It can be estimated by the proportion of observations from the test data set with *Y* = 1 that are correctly predicted:

(8) $$\begin{array}{l} \hat{Se}\left(C_{D_{1}}^{M},D_{t}\right)\\ =\frac{\sum_{i=1}^{T}I\left(y_{t_{i}}=1\right)\cdot I\left(C_{D_{1}}^{M}\left(x_{t_{i}}\right)=1\right)}{\sum_{i=1}^{T}I\left(y_{t_{i}}=1\right)}, \end{array}$$

whereas the *specificity* is the probability *P*(*C*_**D**_**1**__*^M^*(**X**) = 0 | *Y* = 0) of correctly identifying a non-responder and can be estimated by the proportion of observations with *Y* = 0 that are correctly predicted:

(9) $$\begin{array}{l} \hat{Sp}\left(C_{D_{1}}^{M},D_{t}\right)\\ =\frac{\sum_{i=1}^{T}I\left(y_{t_{i}}=0\right)\cdot I\left(C_{D_{1}}^{M}\left(x_{t_{i}}\right)=0\right)}{\sum_{i=1}^{T}I\left(y_{t_{i}}=0\right)}. \end{array}$$

Related useful concepts are the *positive predictive value* and the *negative predictive value*, which depend on the prevalence of the condition *Y* = 1 in the population. It does not make sense to calculate them if the class frequencies for the considered *n* patients are not representative for the population of interest, as is often the case in case-control studies.

#### Decision theoretic aspects

When considering sensitivity and specificity it can be interesting to incorporate the idea of *cost* or *loss* functions from decision theory to evaluate misclassification costs. Instead of the error rate defined in Eq. (7), where a neutral cost function is used implicitly, one could use other cost functions, where the costs and thus the weights in the computation of the error rate, are defined depending of the relative seriousness of misclassifications.

More precisely, a neutral, often referred to as scientific cost function assigns unit costs whenever an observation is misclassified (regardless of the true and predicted class), and no costs when the observation is correctly classified. However, if, for instance, classifying an observation with *Y* = 1 as *Y* = 0 is more serious than vice-versa, such errors should have more weight, i.e. higher misclassification costs. Many classifiers allow to assign such asymmetric misclassification costs, either directly or via class priors. The following principle is obvious for Bayesian methods, where different prior weights may be assigned to the response classes, but also applies to, e.g. classification trees. Imagine that there are much more observations in class 0 than in class 1. Then, in order to reduce the number of misclassifications predicting class 1 for all observations—regardless of the values of the predictor variables—would be a pretty good strategy, because it would guarantee a high number of correctly classified observations.

This principle can be used to train a classifier to concentrate on one class, even if the proportions of class 0 and 1 observations in the actual population and data set are equal: one either has to “make the classifier believe” that there were more observations of class 0 by means of setting a high artificial prior probability for this class, or one has to “tell” the classifier directly that misclassifications of class 0 are more severe by means of specifying higher misclassification costs (cf, e.g. Ripley, 1996). Obviously, such changes in the prior probabilities and costs, that are internally handled as different weights for class 0 and 1 observations, affect sensitivity and specificity. For example, when misclassification of a responder as a non responder is punished more severely than vice-versa, the sensitivity (for correctly detecting a responder) increases, while at the same time the specificity (for correctly identifying a non-responder) decreases, because the classifier categorizes more observations as responders than under a neutral cost scheme.

From a decision theoretic point of view, what we considered as costs so far were actually “regrets” in the sense that the overall costs, e.g. for diagnosing a subject, were not included in our reasoning: only the particular costs induced by a wrong decision were considered, while the costs of correct decisions were considered to be zero. This approach is valid for the comparison of classifiers because the additional costs, e.g. for diagnosing a subject are equal for all classifiers.

#### ROC curves

To account for the fact that the sensitivity and specificity of a classifier are not fixed characteristics, but are influenced by the misclassification cost scheme, the *receiver operating characteristic* (ROC) approach (cf, e.g. Swets, 1988, for an introduction and application examples) could be borrowed from signal detection, and could be used for comparing classifier performance, incorporating the performance under different cost schemes. Then, for each classifier a complete ROC curve describes the sensitivity and specificity under different cost schemes. The curves of two classifiers are directly comparable when they do not intersect. In this case the curve that is further from the diagonal, which would correspond to random class assignment, represents the better classifier. Confidence bounds for ROC curves can be computed (e.g. Schäfer, 1994). The distance from the diagonal, measured as the so called *area under curve* (AUC), is another useful diagnostic (Hanley and McNeil, 1982) and can be estimated via several approaches (e.g. DeLong et al. 1988). As an example, Figure 3 depicts four ROC curves corresponding to different prediction strengths. The diagonal corresponds to an AUC of 0.5 (random assignment), whereas the dotted line yields the optimal value AUC = 1 and the two dashed lines represent intermediate cases as commonly found in practice.

### Credal classification

So far we have considered only the case that the classifier gives a clear class prediction for each observation, say 0 or 1. In addition to this we noted that some classifiers may also return predicted class probabilities instead. Obviously, when the probability for class 1 is, say, 99% we would predict that class without hesitation. However, tree classifiers or ensemble methods that perform majority voting would also predict class 1 when its predicted probability is only, say, 51%—as long as the probability for class 1 is higher than that for class 0, no matter how little the difference. In such a situation one might argue that there should be a third option, namely refusing to predict a class whenever the predicted probability is within a certain threshold or returning the extra value “in doubt” (cf Ripley, 1996, p. 5; 17 f.) if further information is needed to classify an observation.

Several authors have argued along a similar line, for instance the fuzzy set approach by Chianga and Hsub (2002), whose classifier returns the predicted degree of possibility for every class rather than a single predicted class, and Zaffalon (2002), who argues in favor of so-called “credal classification”, where a subset of possible classes for each configuration of predictor variables is returned when there is not enough information to predict one single class (see also Zaffalon et al. 2003, for an application to dementia diagnosis).

After this overview on accuracy measures for the comparison of classifiers, the next section describes possible sampling strategies for the evaluation of accuracy measures. Suggestions on the use of these sampling strategies, as well as a discussion of possible abuses, are given in Section 5.

## 4 Evaluation Strategies

For simplicity, we assume in the following that the error rate is used as an accuracy measure, but the same principles hold for other measures such as the sensitivity or the specificity. The goal of classifier evaluation is the estimation of the conditional error rate *Err* (*C*_**D**_*^M^*) from Eq. (4) or of the unconditional error rate ε_**F**^*n*^_*^M^* (cf Eq. (5)), where the focus on *Err C***D***^M^* or ε_**F**^*n*^_*^M^* depends on the concrete context. For example, a study that aims at designing a classifier based on a particular data set for concrete use in medical practice will focus on *Err* (*C***D***^M^*) rather than ε_**F**^*n*^_*^M^* , whereas a statistical comparison study of classification methods should be as general as possible, and thus focus on the unconditional error rate ε_**F**^*n*^_*^M^* . Readers interested in the difference between conditional and unconditional error rate may refer to Efron and Tibshirani (1997); Molinaro et al. (2005). In general, the question whether unconditional or conditional inference should be preferred is one of the central foundational issues in statistics, where in the frequentist-Bayesian debate the former usually advocate in favor of the unconditional point of view while Bayesian inference is *eo ipso* conditional (cf, e.g. Berger, 1980, Section 1.6). Also a view at the corresponding discussion in sampling theory on evaluating post stratification is illuminating in this context (see, e.g. Hold and Smith, 1979, for a classical paper).

In this article, we arbitrarily use the notation ε̂ for all the estimators, which refers to the unconditional error rate. However, the reviewed estimators can also be seen as estimators of *Err* (*C***D***^M^*). For each method, we denote the estimator in a way that all the quantities influencing it are visible. These expressions, and the corresponding formulas, should be understood as pseudo-code to be used for implementing the procedure.

In addition, all the methods reviewed in the present section are summarized in Table 1.

### Resubstitution

The easiest—and from a statistical point of view by far the worst—evaluation strategy consists of building and evaluating a classifier based on the same data set **Dl**. Usually, the data set **Dl** includes all the available data, i.e. **Dl** = **D**, yielding the estimator

(10) $${\hat{ɛ}}_{RESUB}^{M}(D)=\frac{1}{n}\sum_{i=1}^{n}I\left(y_{i}\neq C_{D}^{M}(x_{i})\right)$$

(11) $$=\hat{Err}\left(C_{D}^{M},D\right).$$

ε̂*_RESUB_^M^* is a downwardly biased estimator of ε_**F**^*n*^_*^M^* and *Err* (*C***D***^M^*), i.e. accuracy is overestimated. Since the constructed classifier *C***D***^M^* was especially designed to fit **D**, it usually performs well on it. The problem of *overfitting*, i.e. that the classifier is too closely adapted to the learning sample, is not specific to microarray data, but it can be enhanced by their high dimensionality: with a huge number of predictor variables, a very subtle partition of the feature space can be achieved, yielding distinct predictions for very small groups of observations. In such a situation it is possible to find a prediction rule such that almost all observations from the learning data set are predicted correctly. However, this does not imply that the prediction rule that is highly adapted to the learning data set will also predict independent new observations correctly.

### Test data set

To evaluate the performance of a classification method on independent observations, one should consider non-overlapping learning and test data sets. A classifier is built based on the learning data set only and subsequently applied to the test observations. If, as above, **l** and **t** contain the indices of the observations included in the learning and test data sets, respectively, the error rate is estimated as

(12) $${\hat{ɛ}}_{TEST}^{M}(D,(l,t))=\frac{1}{T}\sum_{i=1}^{T}I\left(y_{t_{i}}\neq C_{D_{1}}^{M}(x_{t_{i}})\right)$$

(13) $$=\hat{Err}\left(C_{D_{1}}^{M},D_{t}\right),$$

where *T* denotes the size of **t**. In practice, **l** and **t** most often form a partition of {1, *…, n*}, i.e. **t** = {1, *…, n*}\ **l** and ε*_TEST_* *^M^* can be seen as a function of **D** and **l** only. However, we keep the notation as general as possible by including **t** in ε*_TEST_^M^* (**D**, (**l**, **t**)), in order to allow the specification of learning and test sets that do not form a partition of {1, …, *n*} (for instance, when there are two different test data sets). Note that, in contrast to resubstitution, this procedure may have a random component: it depends on the learning and test sets defined by (**l**, **t**). When **l** and **t** are not defined randomly but are chosen by the user (e.g. chronologically where the first recruited patients are assigned to **l** and the following patients to **t**), ε̂*_TEST_* depends on the number of patients in **l**, which is fixed by the user.

Note that, due to the fact that some of the observations from the learning data set are held back for the test set and thus the learning data set contains only *L* < *n* observations, the estimation of the prediction rule from the learning data set is worse and the resulting prediction error increases. Therefore ε̂*_TEST_^M^* has positive bias as an estimator of ε_**F**^*n*^_*^M^* and *Err* (*C^M^***D**), i.e. the obtained prediction accuracy is worse than if all *n* observations were used. This effect does not only occur here, where the original learning data set is split into one learning and test set, but also in the following sections whenever the number of observations in the learning data set is decreased. The 0.632 estimator introduced below addresses this problem. For a discussion of potential changes in the data generating process over time see Section 5.

### Cross-validation

Another option to evaluate prediction accuracy consists of considering all the available observations as test observations successively in a procedure denoted as *cross-validation* (CV), see e.g. Hastie et al. (2001) for an overview. The available observations {1, *…, n*} are divided into *m* non-overlapping and approximately equally sized subsets whose indices are given by **t**^(1)^, *…,* **t**^(^*^m^*^)^. The cross-validation procedure consists of a succession of *m* iterations, hence the name *m*-fold cross-validation. In the *j*-th iteration, the observations defined by **t**^(^*^j^*^)^ are considered as test data and the remaining observations form the learning data set defined by **l**^(^*^j^*^)^ = {1, *…, n*}\**t**^(^*^j^*^)^. The test observations from **Dt**_(_ *_j_*_)_ are then predicted with the classifier *C*_**D**_**1**_*j*)_*^M^*constructed using **D**_**1**^(*j*)^_.

A prediction is thus obtained for each of the *n* observations. The error rate is estimated as the mean proportion of misclassified observations over all cross-validation iterations:

(14) $$\begin{array}{l} {\hat{ɛ}}_{CV}\left(D,{\left(t^{\left(j\right)}\right)}_{j=1,\ldots ,m}\right)\\ =\sum_{j=1}^{m}\frac{n_{t^{\left(j\right)}}}{n}{\hat{ɛ}}_{TEST}^{M}\left(D,(l^{\left(j\right)},t^{\left(j\right)})\right). \end{array}$$

This formula simplifies to

(15) $$\begin{array}{l} {\hat{ɛ}}_{CV}\left(D,{\left(t^{\left(j\right)}\right)}_{j=1,\ldots ,m}\right)\\ =\frac{1}{m}\sum_{j=1}^{m}{\hat{ɛ}}_{TEST}^{M}\left(D,(l^{\left(j\right)},t^{\left(j\right)})\right), \end{array}$$

if **t**^(1)^*, …,* **t**^(^*^m^*^)^ are equally sized. Note that **l**^(^ *^j^*^)^ does not appear as an argument of ε*_CV_^M^*, since **l**^(^ *^j^*^)^ is derived deterministically from **t**^(^ *^j^*^)^ as **l**^(^ *^j^*^)^ = {1*, …, n*}\**t**^(^ *^j^*^)^.

In this setting again decision theoretic considerations could be very helpful, leading to criteria going beyond the mere averaging of misclassified observations. For instance, a more conservative approach inspired by the minimax-decision criterion would be to consider for each classifier the maximum, instead of the average, proportion of misclassified observations over all cross-validation samples and finally choose the classifier with the minimal maximum proportion of misclassified observations over all classifiers. This approach could be called for in situations where not the average or expected performance is of interest but rather it is necessary to guarantee that a certain performance standard is held even in the worst case.

An important special case of cross-validation is *m* = *n*, where **t**^(^ *^j^*^)^ = *j*, i.e. the *n* observations are considered successively as singleton test data sets. This special case is usually denoted as *leave-one-out cross-validation* (LOOCV), since at each iteration one observation is left out of the learning data set. The corresponding error rate estimator can be expressed as

(16) $${\hat{ɛ}}_{LOOCV}^{M}(D)=\frac{1}{n}\sum_{i=1}^{n}{\hat{ɛ}}_{TEST}^{M}\left(D,\left(\{1,\ldots ,n\}\backslash i,i\right)\right).$$

LOOCV is deterministic, in contrast to cross-validation with *m* < *n* which possibly yields different results depending on the (randomly) chosen partition **t**^(1)^*, …,* **t**^(^*^m^*^)^. As an estimator of ε_**F**^*n*^_*^M^* and *Err* (*C***D***^M^*), ε̂*_LOOCV_^M^* (**D**) is almost unbiased, since classifiers are built based on *n –* 1 observations. However, as an estimator of ε_**F**^*n*^_*^M^* , it can have high variance because the learning sets are very similar to each other (Hastie et al. 2001).

In order to reduce the variability of cross-validation results due to the choice of the partition **t**^(1)^, *…,* **t**^(^*^m^*^)^, it has been proposed to average the results of cross-validation obtained for several different partitions. As an example, Braga-Neto and Dougherty (2004) examine what they denote as *CV* 10:

(17) $$\begin{array}{l} {\hat{ɛ}}_{CV10}^{M}\left(D,{\left(t^{(j)k}\right)}_{k=1,\;\ldots ,10,\;\;j=1,\;\ldots ,\;m}\right)\\ =\frac{1}{10}\sum_{k=1}^{10}{\hat{ɛ}}_{CV}\left(D,{\left(t^{(j)k}\right)}_{\;j=1,\;\;\ldots ,\;\;m}\right), \end{array}$$

where (**t**^(1)^*^k^*, *…,* **t**^(^*^m^*^)^*^k^* ) is the partition corresponding to the *k*-th cross-validation. Note that, like, ε̂*_CV_^M^*, the estimator ε̂*_CV_*_10_*^M^* has a random component. However, its variance is reduced by averaging over several partitions.

In stratified cross-vlalidation, each subset **t**^(^*^j^*^)^ contains the same proportion of observations of each class as the whole data set. It is well-established that stratified cross-validation improves the estimation of the error rate.

### Monte-Carlo cross-validation (or subsampling)

Like cross-validation, *Monte-Carlo cross-validation* (MCCV) strategies consist of a succession of iterations and evaluate classification based on test data sets that are not used for classifier construction. It may be seen as an averaging of the test set procedure over several splits into learning and test data sets. In contrast to cross-validation, the test sets are not chosen to form a partition of {1, *…, n*}. In Monte-Carlo cross-validation (also called random splitting or subsampling), the learning sets **l**^(^*^b^*^)^ (*b* = 1, *…, B*) are drawn out of {1, *…, n*} randomly and without replacement. The test sets consist of the remaining observations **t**^(^*^j^* ^)^ = {1, *…, n*} *\***l**^(^*^b^*^)^. The common size ratio *n*_**1**^(*b*)^_ : *n*_**t**^(*b*)^_ is fixed by the user. Usual choices are, e.g. 2 : 1, 4 : 1 or 9 : 1. Each test set contains the observations that are not in the corresponding learning set. The MCCV error rate is given as

(18) $$\begin{array}{l} {\hat{ɛ}}_{MCCV}^{M}\left(D,{\left(l^{\left(b\right)}\right)}_{b=1,\;\ldots ,\;B}\right)\\ =\frac{1}{B}\sum_{b=1}^{B}{\hat{ɛ}}_{TEST}^{M}\left(D,(l^{\left(b\right)},t^{\left(b\right)})\right). \end{array}$$

This formula is identical to the formula of ɛ̂*_CV_^M^* for regular cross-validation, except that the summation is done with respect to the *B* random subsamples and that ɛ̂*_MCCV_^M^* is considered as a function of the learning sets instead of the test sets here for consistency with the bootstrap sampling procedure reviewed in the next section. As an estimator of ɛ_**F**^*n*^_*^M^* , ɛ̂*_MCCV_^M^* has a smaller variance than, e.g. ɛ̂*_LOOCV_^M^*, since it is based on learning sets that are not as highly correlated as those of LOOCV. However, ɛ̂*_MCCV_^M^* is again upwardly biased as an estimator of both ɛ_**F**^*n*^_*^M^* and *Err* (*C***D***^M^*), i.e. accuracy is underestimated, since the prediction rules are constructed based on less than *n* observations.

### Bootstrap sampling

In bootstrap sampling, the learning sets l^*^^(^*^b^*^)^ are drawn out of {1*, …, n*} randomly and with replacement. The * symbol indicates that each observation may be represented several times in l^*^^(^*^b^*^)^. The (common) size of the learning sets is most often set to *n*. Hence, each l^*^^(^*^b^*^)^ includes an average of 1 *−* (1 *−* 1*/n*)*^n^* ≈_→ ∞ n_ 63.2% of the *n* observations at least once. The test sets **t**^(^*^b^*^)^ are again formed by the observations which are not in the corresponding learning set l^*^^(^*^b^*^)^. Note that each test may have a different number of observations. In each of the *B* bootstrap iterations, the learning data set is used to construct a classifier *C*_**D**_1*_^(*b*)^_*^M^* that is subsequently applied to the test set **D**_**t**(*j*)_. There are several variants for estimating the error rate based on these results. The first variant consists of considering all the predictions simultaneously and computing the global error as

(19) $$\begin{array}{l} {\hat{ɛ}}_{BOOT1}^{M}\left(D,{\left(l^{*(b)}\right)}_{b=1,\;\ldots ,\;B}\right)\\ =\frac{\sum_{i=1}^{n}\sum_{b=1}^{B}I_{i}^{\left(b\right)}\cdot I\left(y_{i}\neq C_{D_{1{*}^{\left(b\right)}}}^{M}(x_{i})\right)}{\sum_{i=1}^{n}\sum_{b=1}^{B}I_{i}^{\left(b\right)}}, \end{array}$$

with

*I_i_*^(^*^b^*^)^ = 0 if observation *i* is included in the learning set l^*^^(^*^b^*^)^ at least once,

= 1 else.

Note that the MCCV error estimator presented in the previous section may also be expressed in this way. In contrast, the second bootstrap variant considers each observation individually and estimates the error rate as

(20) $${\hat{ɛ}}_{BOOT2}^{M}\left(D,{\left(l^{*(b)}\right)}_{b=1,\;\ldots ,\;B}\right)=\frac{1}{n}\sum_{i=1}^{n}{\hat{E}}_{i},$$

where *Ê_i_* is the averaged individual error rate of observation *i* over the iterations:

$${\hat{E}}_{i}=\frac{\sum_{b=1}^{B}I_{i}^{\left(b\right)}\cdot I\left(y_{i}\neq C_{D_{1{*}^{\left(b\right)}}}^{M}(x_{i})\right)}{\sum_{b=1}^{B}I_{i}^{\left(b\right)}}.$$

These two variants agree when *B →* 0 and usually produce nearly identical results (Efron and Tibshirani, 1997).

Note that the principle of bootstrap learning samples that determine their own test samples (the observations not included in the current bootstrap sample, also called *“out-of-bag” observations*) is also incorporated in the recent ensemble methods *bagging* (Breiman, 1996) and *random forests* (Breiman, 2001). Here the prediction accuracy of ensembles of classifiers learned on bootstrap samples is evaluated internally on the out-of-bag observations. Therefore these methods have a built-in control against overoptimistic estimations of the error rate.

### The 0.632 and 0.632+ estimators

Bootstrap estimators of the error rate are upwardly biased, since classifiers are built using on average only 63.2% of the available observations. That is why Efron and Gong (1983) suggest an estimation procedure that combines the bootstrap sampling error rate and the resubstitution error rate. They define the 0.632 *estimator* as

(21) $$\begin{array}{l} {\hat{ɛ}}_{0.632}^{M}\left(D,{\left(l^{*(b)}\right)}_{b=1,\;\ldots ,\;B}\right)\\ =0.368\;\cdot \;{\hat{ɛ}}_{RESUB}^{M}(D)\\ +0.632\;\cdot \;{\hat{ɛ}}_{BOOT1}\left(D,{\left(l^{*(b)}\right)}_{b=1,\;\ldots ,\;B}\right), \end{array}$$

which is designed to correct the upward bias in ɛ̂*_BOOT_*_1_ by averaging it with the downwardly biased resubstitution error rate ɛ̂*_RESUB_^M^* . The 0.632+ estimator is suggested by Efron and Tibshirani (1997) as a less biased compromise between resubstitution and bootstrap errors designed for the case of strongly overfitting classifiers. These estimates have lower bias than MCCV or simple bootstrap sampling estimates. Their principle is generalized to survival prediction by Gerds and Schumacher (2007).

### Bootstrap cross-validation

Fu et al. (2005) suggest an approach denoted as bootstrap cross-validation combining bootstrap estimation and LOOCV. The resulting error rate estimator can be seen as a bagging predictor, in the sense that the final error rate estimate results from the combination of several (LOOCV) estimates based on bootstrap samples. For each of the *B* bootstrap samples, LOOCV is carried out. Error estimation is then obtained by averaging the LOOCV result over the *B* bootstrap iterations.

Since bootstrap samples have duplicates, learning and test sets may overlap for the corresponding CV iterations. Fu et al. (2005) claim that such an overlapping should be seen as an advantage rather than a disadvantage for small samples, since correcting the upward bias of bootstrap error estimation. Bootstrap cross-validation is reported to perform better than bootstrap and the 0.632 and 0.632+ estimators (Fu et al. 2005).

In this section, we have mainly focused on the potential bias of error rate estimation approaches. The estimation of the variance of these estimators, which is a very complex task in the case of small samples, is addressed in several recent articles. For instance, Berrar et al. (2006) consider the problem of comparisons between several error rates (involving a multiple testing component) and confidence intervals for error rates, whereas Wickenberg-Bolin et al. (2006) suggest an improved variance estimation method for small sample situations. Another aspect to be considered when interpreting error rates is the comparison to error rates obtained using different variants of trivial random classifiers, see Wood et al. (2007) for a thorough discussion of this topic.

## 5 Which Evaluation Scheme in Which Situation?

The evaluation of classification methods may have various goals. One goal may be to compare several classification methods from a methodological point of view and explain observed differences (for instance, Dudoit et al. 2002; Romualdi et al. 2003; Statnikov et al. 2005). Medical or biological articles on the other hand are concerned with the performance *on future independent data* of the best classifier, which should be selected following a strict procedure (typically one of those used in the comparison studies mentioned above).

For that selection procedure, resubstitution should never be employed, since yielding far too optimistic estimates of accuracy. Even if the goal is to compare different methods rather than to estimate the absolute prediction accuracy, resubstitution turns out to be inappropriate, since artificially favoring those methods that overfit the learning data. Hence, an inescapable rule is that classifiers should not be evaluated only on the same data set they were trained on.

In this context, the above warning should be repeated. A classical flaw encountered in the literature consists of selecting variables based on the whole data set and building classifiers based on this reduced set of variables. This approach should be banned, see Ambroise and McLachlan (2002); Simon et al. (2003); Wood et al. (2007) for studies on this topic. Even (and especially) when the number of variables reaches several tens of thousands, variable selection must be carried out for each splitting into learning and test data sets successively.

### Cross-validation, Monte-Carlo cross-validation and bootstrap for classifiers comparison

In a purely statistical study with focus on the comparison of classification methods in high dimensional settings, it is not recommended to estimate prediction accuracy based on a single learning data set and test data set, because for limited sample sizes the results depend highly on the chosen partition (cf, e.g. Hothorn et al. 2005). From a statistical point of view, when the original learning data set is split into one learning and one test set, increasing the size of the test set decreases the variance of the prediction accuracy estimation. However, it also decreases the size of the leftover learning data set and thus increases the bias, since using less observations than available for learning the prediction rule yields an artificially high and variable error. In the case of a very small *n*, this might even lead to the too pessimistic conclusion that gene expression data do not contribute to prediction. Procedures like cross-validation, Monte-Carlo cross-validation or bootstrap sampling may be seen as an attempt to decrease the estimation bias by considering larger learning sets, while limiting the variability through averaging over several partitions into learning and test data sets.

Contradicting studies have been published on the comparison of CV, MCCV and bootstrap strategies for error rate estimation. The use of CV (Eq. (14)) in small sample settings is controversial (Braga-Neto and Dougherty, 2004) because of its high variability compared to MCCV (Eq. (18)) or bootstrap sampling (Eq. (19–20)). For instance, in the case of *n* = 30, each observation accounts for more than 3% in the error rate estimation. For a data set in which, say, at most three patients are difficult to classify, CV does not allow a fair comparison of classification methods. Braga-Neto and Dougherty (2004) recommend bootstrap strategies or repeated CV (denoted as CV10 in the present article, see Eq. (17)) as more robust alternatives. In contrast, another study by Molinaro et al. (2005) taking small sample size and high-dimensionality into account reports good performance for LOOCV estimation, as well as for 5- and 10-fold CV. The low bias of LOOCV, its conceptual simplicity as well as the fact that it does not have any random component make it popular in the context of microarray data. Meanwhile, it has become a standard measure of accuracy used for comparing results from different studies. However, if one wants to use CV, a more recommendable approach consists of repeating cross-validation several times, i.e. with different partitions **t**^(1)^, …, **t**^(^*^m^*^)^, when *m* can take the values, e.g. *m* = 5 or *m* = 10. Averaging over several partitions reduces the variance associated with cross-validation (Braga-Neto and Dougherty, 2004).

Stable estimates of prediction accuracy can also be obtained via MCCV or bootstrap sampling. In MCCV, the choice of the ratio *n***l** : *n***t** might depend on the goal of the study. If the goal is comparison only, a ratio like 2 : 1 may be appropriate. If one is not only interested in the relative performance of the methods but also in the value of the prediction accuracy itself, larger learning sets are conceivable. However, for both CV and MCCV/bootstrap, it must be recalled that the estimate of prediction accuracy *always* tends to be pessimistic compared to the prediction accuracy that would be obtained based on the *n* observations, since less than *n* observations are used for classifier construction. Less biased estimators such as 0.632+ are recommended if the absolute value of the error rate is of importance. However, a very recent study points out a possible bias of bootstrap procedures in favor of more complex prediction models (Binder and Schumacher, 2007), which may affect the comparison of methods.

When on the other hand the aim of a benchmark study is a complete ranking of all considered classifiers with respect to any performance measure the Bradley-Terry(-Luce) model for paired comparisons (Bradley and Terry, 1952) or the recent approach of Hornik and Meyer (2007) for consensus rankings are attractive. In addition to the purely descriptive ranking of these approaches statistical inference on the performance differences between classifiers can be conducted when the test samples are drawn appropriately, e.g. when several CV- or bootstrap-samples are available (Hothorn et al. 2005).

### Validation in medical studies

In medical studies, the problem is different. Investigators are not interested in the methods themselves but in their practical relevance and validity for future independent patient data. The addressed questions are:

1. Can reliable prediction be performed for new patients?
2. Which classification method should be used on these new data?

Whereas the second question is basically the same as in statistical studies, the first question is most often ignored in statistical papers, whose goal is rather to compare methods from a theoretical point of view than to produce “ready-to-use” classifiers to be used in medical practice.

Question 1 can be answered reliably only based on several, or one large, validation data set that has been made available to the statistician after construction and selection of an appropriate classifier. A validation set that remains unopened until the end of the analysis is necessary, in the vein of the validation policy developed by the Sylvia Lawry Centre for Multiple Sclerosis Research (Daumer et al. 2007).

#### Choice of the validation data set

The impact of the reported classifier accuracy in the medical community increases with the differences between validation data set and open data set. For example, it is much more difficult to find similar results (and thus much more impressing when such results are found) on a validation data set collected in a different lab at a different time and for patients with different ethnic, social or geographical background than in a validation set drawn at random from a homogeneous data set at the beginning of the analysis. An important special case is when the learning and validation sets are defined chronologically. In this scheme, the first recruited patients are considered as learning data and used for classifier construction and selection *before* the validation data set is collected, hence warranting that the validation data remain unopened until the end of the learning phase. Obviously, evaluating a classifier on a validation data set does not provide an estimate of the error rate which would be obtained if both learning and validation data set were used for learning the classifier. However, having an untouched validation data set is the only way to simulate prediction of new data. See Simon (2006) for considerations on the validation problem in concrete cancer studies and Buyse et al. (2006) for details on the validation experiments conducted to validate the well-known 70-gene signature for breast cancer outcome prediction by (van’tVeer et al. 2002).

Furthermore, if the learning and test sets are essentially different (e.g. from a geographical or technical point of view), bad performance may be obtained even with a classifier that is optimal with respect to the learning data. The error rate on the validation set increases with i) the level of independence between *Y* and **X** in both learning and validation sets, ii) the difference between the joint distribution **F** of *Y* and **X** in the learning and validation sets, iii) the discrepancy between the optimal Bayes classifier and the constructed classifier. Whereas the components i) and iii) are common to all methods of accuracy estimation, component ii) is specific to validation schemes in which “validation patients” are different from “learning patients”.

Thus, it does make a difference whether the learning and test sets are (random) samples from the same original data set, or if the test set is sampled, e.g. in a different center in a multi-center clinical trial or at a different point in time in a long-term study. The first case—ideally with random sampling of the learning and test sets—corresponds to the most general assumption for all kinds of statistical models, namely the “i.i.d.” assumption that all data in the learning and test sets are randomly drawn independent samples from the same distribution and that the samples only vary randomly from this distribution due to their limited sample size. This common distribution is often called the data generating process (DGP). A classifier that was trained on a learning sample is supposed to perform well on a test sample from the same DGP, as long as it does not overfit.

A different story is the performance of a classifier learned on one data set and tested on another one from a different place or time. If the classifier performs badly on this kind of test sample this can have different reasons: either important confounder variables were not accounted for in the original classifier, e.g. an effect of climate when the classifier is supposed to be generalized over different continents (cf Altman and Royston, 2000, who state that models may not be “transportable”), or—even more severe for the scientist—the DGP has actually changed, e.g. over time, which is an issue discussed as “data drift”, “concept drift” or “structural change” in the literature. In this latter case, rather than discarding the classifier, the change in the data stream should be detected (Kifer et al. 2004) and modelled accordingly—or in restricted situations it is even possible to formalize conditions under which some performance guarantees can be proved for the test set (Ben-David et al. 2007).

When on the other hand the ultimate goal is to find a classifier that is generalizable to all kinds of test sets, including those from different places or points in time, as a consequence we would have to follow the reasoning of “Occam’s razor” for our statistical models: the sparsest model is always the best choice other things being equal. Such arguments can be found in Altman and Royston (2000) and, more drastically, in Hand (2006), who uses this argument not only with respect to avoiding overfitting and the inclusion of too many covariates, but also, e.g. in favor of linear models as opposed to recursive partitioning, where it is, however, at least questionable from our point of view, if the strictly linear, parametric and additive approach of linear models is really more “sparse” than, e.g. simple binary partitioning.

#### Recommendations

With respect to the first question posed at the beginning of this subsection we therefore have to conclude that there are at least one clinical and one—if not a dozen—statistical answers, while for the second question we have a clear recommendation. Question 2 should be addressed based on the open learning data set only via cross-validation, repeated cross-validation, Monte-Carlo cross-validation or bootstrap approaches. The procedure is as follows:

1. Define *N_iter_* pairs of learning and test sets (**1**^(^*^j^*^)^, **t**^(^ *^j^*^)^), *j* = 1 *, …, N_iter_* following one of the evaluation strategies described in Section 4 (LOOCV, CV, repeated CV, MCCV, bootstrap, etc). For example, in LOOCV, we have *N_iter_* = *n*.
2. For each iteration ( *j* = 1 *, …, N_iter_*), repeat the following steps:
  - Construct classifiers based on **l**^(^ *^j^*^)^ using different methods *M*_1_,*M*_2_,...,*M_q_* successively, where *M_r_* (*r* = 1*, …, q*) is defined as the combination of the variable selection method (e.g. univariate Wilcoxon-based variable selection), the number of selected variables (e.g. *p̃* = 50, 100, 500) and the classification method itself (e.g. linear discriminant analysis).
  - Predict the observations from the test set **t**^(^ *^j^*^)^ using the constructed classifiers *C*_**D**_**1**_(*j*)_^*M*^1^^,…, *C*_**D**_**1**_(*j*)_^*M*^*q*^^ successively.
3. Estimate the error rate based on the chosen procedure for all methods *M*_1_, …, *M_q_* successively.
4. Select the method yielding the smallest error rate. It should then be validated using the observations from the independent validation set.

A critical aspect of this procedure is the choice of the “candidate” methods *M*_1_, *…, M_q_*. On the one hand, trying many methods increases the probability to find a method performing better than the other methods “by chance”. On the other hand, obviously, increasing the number of methods also increases the chance of finding the right method, i.e. the method that best reflects the true data structure and is thus expected to show good performance on independent new data as well.

CV, MCCV or bootstrap procedures might also be useful in medical studies for accuracy estimation, but their results should not be over-interpreted. They give a valuable preview of classifier accuracy when the collected data set is still not large enough for putting aside a large enough validation set. In this case, one should adopt one the following approaches for choosing the method parameters:

- Using the default parameter values.
- Selecting parameter values by internal cross-validation (or a related approach) within each iteration (Varma and Simon, 2006). The computational complexity is then *n*^2^, which makes it prohibitive if the chosen classification method is not very fast, especially when it involves variable selection.
- Selecting parameter values based on solid previous publications analyzing other data sets.

Trying several values and reporting only the error rate obtained with the optimal value is an incorrect approach. Studies and discussions on the bias induced by this approach can be found in Varma and Simon (2006); Wood et al. (2007). In all cases, it should be mentioned that such an analysis does not replace an independent validation data set.

## 6 Summary and Outlook

For fair evaluation of classifiers, the following rules should be taken into account.

- The constructed classifier should ideally be tested on a independent validation data set. If impossible (e.g. because the sample is too small), the error rate should be estimated with a procedure which tests the classifier based on data that were not used for its construction, such as cross-validation, Monte-Carlo cross-validation or bootstrap sampling.
- Variable selection should be considered as a step of classifier construction. As such, it should be carried out using the learning data only.
- Whenever appropriate, sensitivity and specificity of classifiers should be estimated. If the goal of the study is, e.g. to reach high sensitivity, it is important to design the classifier correspondingly.

Note that both the construction and the evaluation of prediction rules have to be modified if the outcome is not, as assumed in this paper, nominal, but ordinal, continuous or censored. While ordinal variables are very difficult to handle in the small sample setting and thus often dichotomized, censored survival variables can be handled using specific methods coping with the *n* ≪ *p* setting. Since censoring makes the use of usual criteria like the mean square error impossible, sophisticated evaluation procedures have to be used, such as the Brier score (see VanWieringen et al. (2007) for a review of several criteria).

Another aspect that has not been treated in the present paper because it would have gone beyond its scope is the stability of classifiers and classifier assessment. For instance, would the same classifier be obtained if an observation were removed from the data set? How does an incorrect response specification affect the classification rule and the estimation of its error rate? Further research is needed to answer these most relevant questions, which affect all microarray studies.

Further research should also consider the fact that due to the many steps involved in the experimental process, from hybridization to image analysis, even in high quality experimental data severe measurement error may be present (see, e.g. Rocke and Durbin, 2001; Tadesse et al. 2005; Purdom and Holmes, 2005). As a consequence, prediction and diagnosis no longer coincide, since prediction is usually still based on the mismeasured variables, while diagnosis tries to understand the material relations between the true variables. While several powerful procedures to correct measurement error are available for regression models (see, e.g. Wansbeek and Meijer, 2000; Cheng and Ness, 1999; Carroll et al. 2006; Schneeweiß and Augustin, 2006, for surveys considering linear and nonlinear models, respectively), in the classification context well-founded treatment of measurement error is still in its infancy.

A further problem which is largely ignored by many statistical articles is the incorporation of clinical parameters into the classifier and the underlying question of the additional predictive value of gene expression data compared to clinical parameters alone. Although “adjustment for other classic predictors of the disease outcome [is] essential” (Ntzani and Ioannidis, 2003), this problem is largely ignored by most methodological articles. Specific evaluation and comparison strategies have to be developed to answer this question.

## Figures and Tables

**Figure 1:**
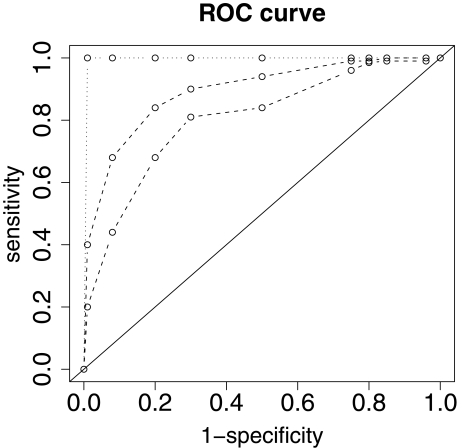
Examples of ROC curves.

**Table 1:** Summary of the reviewed evaluation strategies. **Iterations:** number of iterations, i.e. number of times a classifier is constructed and applied to data; u.d.**=** user-defined. **Bias:** Bias of the error estimation; ↑ means positive bias, i.e. underestimation of prediction accuracy and vice-versa. **Principle:** Gives the definition of the learning and test sets or the used combination of methods.

	Iterations	Bias	Principle
Resubstitution	1	↓	**l** = **t** = {1, …, *n*}
Test	1	↑	{**l**, **t**} from a partition of {1, …, *n*}
LOOCV	*n*	*–*	**t**^(^*^j^*^)^ = {*j* }, **l**^(^*^j^*^)^ = {1, …, *n*}\{*j* },for *j* = 1, …, *n*
m-fold-CV	*m*	↑	**t**^(1)^, …, **t**^(^*^m^*^)^ from a partition of {1, …, *n*} **l**^(^*^j^*^)^ = {1, …, *n*}\ **t**^(^*^j^*^)^, for *j* =1, …, *m*}
MCCV	B (u.d.)	↑	{**l**^(^*^b^*^)^, **t**^(^*^b^*^)^} from a partition of {1, …, *n*}, for *b* = 1, …, *B*
Bootstrap	B (u.d.)	↑	l^*(^*^b^*^)^ is a bootstrap sample drawn out of {1, …, *n*} t^*(^*^b^*^)^*=* {1, …, *n*}\l^*(^*^b^*^)^, for *b* = 1, …, *B*
0.632,0.632+	B (u.d.)	*–*	Weighted sum of resubstitution and bootstrap error rates.
Bootstrap-CV	*n*B (u.d)	*–*	LOOCV within *B* bootstrap samples.

## Appendix A

### Overview of software implementing classification methods in R

Most methods for microarray-based classification are implemented in R (www.R-project.org) which has become the standard statistical tool for handling high-dimensional genomic data. Simple univariate variable selection might be performed, e.g. based on the t-test (t.test) or the Mann-Whitney test (wilcox.test). Usual classifiers like logistic regression (R function glm), linear discriminant analysis (R function lda), quadratic discriminant analysis (R function qda) are also accessible in R without loading any particular package. The same holds for PCA dimension reduction (R function prcomp). Here is a list of specific R packages that are of particular interest for microarray-based classification and freely available without registration.

- pamr package for PAM (Tibshirani et al. 2002)
- penalized package for penalized regression approaches: LASSO, *L*_2_ (Goeman, 2007)
- glmpath package for LASSO regression (Park and Hastie, 2007)
- rda package for regularized discriminant analysis (Guo et al. 2007)
- plsgenomics package for PLS-based classification (Boulesteix, 2004; Fort and Lambert-Lacroix, 2005)
- gpls package for generalized partial least squares classification (Ding and Gentleman, 2005)
- e1071 package for SVM
- randomForest for random forests classification (Diaz-Uriarte and de Andrés, 2006)
- logitBoost package for logitBoost (Dettling and Bühlmann, 2003)
- BagBoosting package for bagboosting (Dettling, 2004)
- MADE4 package for classification by the “between-group analysis” (BGA) dimension reduction method (Culhane et al. 2005)
- pdmclass package for classification using penalized discriminant methods (Ghosh, 2003)
- MLInterfaces package including unifying functions for cross-validation and validation on test data in combination with various classifiers
- MCRestimate package for fair comparison and evaluation of classification methods (Ruschhaupt et al. 2004)

Packages including functions for gene selection are

- genefilter package including a function that computes t-tests quickly
- WilcoxCV package for fast Wilcoxon based variable selection in cross-validation (Boulesteix, 2007)
- varSelRF R package for variable selections with random forests (Diaz-Uriarte and de Andrés, 2006)
- GALGO R package for variable selection with genetic algorithms (Trevino and Falciani, 2006) (http://www.bip.bham.ac.uk/vivo/galgo/AppNotesPaper.htm).
- MiPP package to find optimal sets of variables that separate samples into two or more classes (Soukup and Lee, 2004; Soukup et al. 2005)

## Appendix B

### Summary of six comparison studies of classification methods

**Table 2:** Summary of six comparison studies of classification methods. This summary should be considered with caution, since not detailing the used variants of the considered methods.

**Dudoit et al. (2002)** 3 data sets MCCV 2:1	Included: LDA, DLDA, DQDA, Fisher, *k*NN, trees, tree-based ensembles Variable selection: F-statistic *Conclusion*: DLDA and *k*NN perform best
**Romualdi et al. (2003)** 2 data sets CV	Included: DLDA, trees, neural networks SVM, *k*NN, PAM combined with: Variable selection/dimension reduction: PLS, PCA, soft thresholding, GA/*k*NN *Conclusion*: PLS transformation is recommendable, No classifier uniformly better than the other
**Man et al. (2004)** 6 data sets LOOCV, bootstrap	Included: *k*NN, PCA+LDA, PLS-DA, neural networks, random forests, SVM Variable selection: F-statistic *Conclusion*: PLS-DA and SVM perform best
**Lee et al. (2005)** 7 data sets LOOCV, MCCV 2:1	Included: 21 methods (e.g. tree ensembles, SVM, LDA, DLDA, QDA, Fisher, PAM) Variable selection: F-statistic, rank-based score, soft thresholding *Conclusion*: No classifier uniformly better than the other, rank-based variable selection performs best
**Statnikov et al. (2005)** 11 data sets LOOCV, 10-fold CV	Included: SVM, *k*NN, probabilistic neural networks, backpropagation neural networks Variable selection: BSS/WSS, Golub et al. (1999), Kruskal-Wallis test *Conclusion*: SVM performs best
**Huang et al. (2005)** 2 data sets LOOCV	Included: PLS, penalized PLS, LASSO, PAM, random forests Variable selection: F-statistic Random forests perform slightly better *Conclusion*: No classifier uniformly better than the other
